# Deciphering Sex-Specific Differentiation of Human Fetal Gonads: Insight From Experimental Models

**DOI:** 10.3389/fcell.2022.902082

**Published:** 2022-06-02

**Authors:** Malene Lundgaard Riis, Anne Jørgensen

**Affiliations:** ^1^ Department of Growth and Reproduction, Copenhagen University Hospital—Rigshospitalet, Copenhagen, Denmark; ^2^ International Research and Research Training Centre in Endocrine Disruption of Male Reproduction and Child Health (EDMaRC), Copenhagen University Hospital—Rigshospitalet, Copenhagen, Denmark

**Keywords:** human fetal gonads, sex-specific gonadal differentiation, differences/disorders of sex development, *ex vivo* culture models, fetal testis development, fetal ovary development

## Abstract

Sex-specific gonadal differentiation is initiated by the expression of SRY in male foetuses. This promotes a signalling pathway directing testicular development, while in female foetuses the absence of SRY and expression of pro-ovarian factors promote ovarian development. Importantly, in addition to the initiation of a sex-specific signalling cascade the opposite pathway is simultaneously inhibited. The somatic cell populations within the gonads dictates this differentiation as well as the development of secondary sex characteristics via secretion of endocrine factors and steroid hormones. Opposing pathways SOX9/FGF9 (testis) and WNT4/RSPO1 (ovary) controls the development and differentiation of the bipotential mouse gonad and even though sex-specific gonadal differentiation is largely considered to be conserved between mice and humans, recent studies have identified several differences. Hence, the signalling pathways promoting early mouse gonad differentiation cannot be directly transferred to human development thus highlighting the importance of also examining this signalling in human fetal gonads. This review focus on the current understanding of regulatory mechanisms governing human gonadal sex differentiation by combining knowledge of these processes from studies in mice, information from patients with differences of sex development and insight from manipulation of selected signalling pathways in *ex vivo* culture models of human fetal gonads.

## Brief Overview of Sex-specific Gonadal Differentiation in Mice

Currently, the understanding of mechanisms directing sex-specific cell fate decisions in human bipotential gonads in male or female direction is largely derived from studies in mice. It is generally considered that these processes are largely conserved between mice and humans although there appear to be some exceptions to this notion. With the use of knockout mouse models, the signalling pathways and mechanisms controlling sex-specific differentiation of the bipotential gonad has been extensively studied in recent years (reviewed in detail in Wilhelm et al., 2007; Greenfield, 2015; Lin and Capel, 2015; Rotgers et al., 2018; Yang et al., 2018; Capel, 2019; Nef et al., 2019; Yildirim et al., 2020)). In brief, the presence of the Sex-determining Region Y (SRY) gene, expressed from the Y-chromosome at embryonic day (E) 10.5, initiates Sertoli cell differentiation from the supporting cell lineage (Koopman et al., 1990; Bullejos and Koopman, 2001). This is mediated through upregulation of SRY-related High Mobility Group (HMG) box 9 (SOX9) (da Silva et al., 1996; Sekido et al., 2004), and failure to initiate *Sox9* expression in XY gonads results in male-to-female sex reversal (Gonen et al., 2018). Likewise, induction of *Sox9* expression in XX gonads results in testicular development (Bishop et al., 2000; Vidal et al., 2001). Downstream of SOX9, Fibroblast Growth Factor 9 (FGF9) contribute to the early sex-specific differentiation of the Sertoli cells (Schmahl et al., 2004), and knockout of either FGF9 or its associated receptor Fibroblast Growth Factor Receptor 2 (FGFR2) results in male-to-female sex reversal (Colvin et al., 2001; DiNapoli et al., 2006; Kim et al., 2007). The Sertoli cells in the fetal testis secrete Anti-Müllerian Hormone (AMH) that ensures Mullerian duct regression (Behringer et al., 1990), as well as Desert Hedgehog(DHH) and Platelet Derived Growth Factor (PDGF), which promotes the differentiation and maturation of the fetal Leydig cells (Yao et al., 2002; Brennan et al., 2003). Accordingly, loss of *Dhh* results in impaired Leydig cell differentiation and feminization of male mice (Clark et al., 2000), while inhibition of DHH signalling in *ex vivo* cultured fetal mouse testes disrupt Leydig cell differentiation (Yao and Capel, 2002).

In XX gonads, absence of SRY in combination with the expression of pro-ovarian factors Wingless-related MMTV Integration Site 4 (WNT4), Rspondin-1 (RSPO1) and downstream *β*-catenin promotes the differentiation of granulosa cells. This subsequently reinforce the female fate decision in all the other cell types present in the gonad, thus promoting development of the ovary. Loss-of-function of either of these regulators result in various degrees of gonadal dysgenesis in XX gonads. Importantly, knockout of *Rspo1* in XX gonads results in masculinization of the gonads with testis-like vasculature, increased expression of steroidogenic enzymes and increased testosterone production (Chassot et al., 2008; Tomizuka et al., 2008). Similarly, knockout of *Wnt4* in XX gonads results in increased testosterone biosynthesis and partial female-to-male sex reversal (Vainio et al., 1999; Tang et al., 2020). Disruption of *β*-catenin signalling in the somatic cells of XX gonads also result in masculinization of gonads (Liu et al., 2009), thus resembling the phenotype observed in ovaries with knockout of *Wnt4* or *Rspo1*. Another important factor involved in the promotion of ovarian development is Forkhead Box L2 (FOXL2), which plays a role in establishing the granulosa cells during fetal development and in maintaining cell identity in the adult mice (Schmidt et al., 2004; Ottolenghi et al., 2005; Uhlenhaut et al., 2009). Although FOXL2 is not considered essential for the early sex-specific differentiation of the granulosa cells, overexpression in XY gonads results in impaired seminiferous cord structures and reduced AMH expression (Ottolenghi et al., 2007). In comparison to the prenatal differentiation of fetal Leydig cells, the differentiation of steroidogenic cell lineage precursors to theca cells occur postnatally in mice ovaries which also relies to some extent on Hedgehog signalling (Cowan and Quirk, 2021).

Importantly, sex-specific gonadal differentiation in both sexes involves the continuous repression of the opposite pathway which is crucial to ensure maintenance of the initially established fate of the gonad. Several studies have shown failure to maintain gonadal cell fate decision in adult mice following loss of the antagonistic actions of FOXL2 and Doublesex And Mab-3 Related Transcription Factor 1 (DMRT1) resulting in trans-differentiation of granulosa and Sertoli cells, respectively (Uhlenhaut et al., 2009; Matson et al., 2011; Lindeman et al., 2015).

Another fundamental difference in the early development of ovaries and testes are seen in the timing of germ cell meiotic entry that is controlled by the somatic niche. Meiosis is initiated between E13.5 and E15.5 in germ cells of the fetal ovary and it has been suggested to involve the action of retinoic acid (RA) through up-regulation of the pre-meiosis factor *Stra8* (Baltus et al., 2006; Bowles et al., 2006, 2016; Koubova et al., 2006; Tedesco et al., 2013). However, the involvement of RA in the initiation of meiosis has been extensively debated since several studies have found RA dispensable for meiotic entry (Kumar et al., 2011; Chassot et al., 2020; Vernet et al., 2020). In the initial studies exogenous RA resulted in up-regulation of *Stra8* in both sexes (Bowles et al., 2006), while treatment with RA receptor (RAR) antagonists was shown to inhibit the induction of *Stra8* expression and reduce the number of meiotic germ cells in fetal ovaries (Bowles et al., 2006; Koubova et al., 2006). Additionally, culture of fetal testis with RAR agonists induced premature expression of *Stra8* (Koubova et al., 2006). In accordance, meiosis was delayed when the gene for the RA-synthesizing enzyme *Aldh1a1* was knocked out in fetal ovaries (Bowles et al., 2016). However, subsequent studies have showed that deletion of RA-producing enzymes by double-knockout (*Aldh1a2/3*) or triple-knockout (*Aldh1a1/2/3*) does not entirely ablate *Stra8* expression and induction of meiosis in fetal ovaries (Kumar et al., 2011; Chassot et al., 2020). Similar results were also found when all three RARs were deleted (Vernet et al., 2020). Thereby suggesting that RA signaling is not required for the initiation of meiosis. Despite these results, a recent study then showed that RA does induce *Stra8* expression through retinoic acid responsive elements (RARE) and targeted mutations in these elements demonstrated that they are required for full *Stra8* expression (Feng et al., 2021). Thus, the involvement of RA in the promotion of meiotic entry remains debated and several reviews have specifically discussed this (Kumar et al., 2013; Yadu and Kumar, 2019; Schleif, Havel and Griswold, 2022; Spiller and Bowles, 2022). Interestingly, recent single cell RNA sequencing data have shown that germ cells display a sex-specifically divergent transcriptional pattern as early as E11.5 with upregulation of the Nodal/Activin pathway observed in XY gonads (Mayère et al., 2021). Conversely, in XX germ cells the Bone Morphogenic Protein (BMP) signalling pathway is upregulated (Mayère et al., 2021), and together with another recent study demonstrating that downstream BMP signalling protein ZGLP1 as determinant of oogenic fate and meiotic entry (Nagaoka et al., 2020), this suggest a role for the BMP signalling pathway in regulating female germ cell differentiation.

Conversely, in fetal testes inhibitory signals from the Sertoli cells ensure that meiosis is actively repressed in the testis until around postnatal day 8–10 (Nebel et al., 1961). This repression has been suggested to involve the RA-degrading enzyme CYP26B1 (Bowles et al., 2006; Koubova et al., 2006) as germ cells in testes of *Cyp26b1*-knockout mice upregulates *Stra8* expression and enter meiosis prematurely (Bowles et al., 2006). However, since opposing evidence exist related to the role of RA as inducer of meiosis, it remains to be elucidated whether CYP26B1 has other functions in the fetal testis than the suggested function of RA degradation (Bowles et al., 2006, Bowles et al., 2016; Koubova et al., 2006; Kumar et al., 2011; Chassot et al., 2020; Vernet et al., 2020). Importantly, several other factors including DMRT1 (Matson et al., 2010; Minkina et al., 2014), NANOS2 (Suzuki and Saga, 2008), FGF9 (Bowles et al., 2010) and Nodal (Souquet et al., 2012; Wu et al., 2013) have been shown to be involved in repression of meiotic entry in the fetal testis.

## Development and Sex-Specific Differentiation of the Human Fetal Bipotential Gonad

In humans, the bipotential gonads develop from around gestational week (GW) 4 where the genital ridges appear as thickenings of the intermediate mesoderm (Byskov, 1986). Proliferation of the overlying coelomic epithelium give rise to the somatic cell populations of the forming gonads. Coinciding with the appearance of the genital ridges, primordial germ cells (PGC) migrate from the proximal epiblast via the hindgut to the developing gonads, where they arrive during GW 5 (Fujimoto et al.,, 1977; Mollgard et al., 2010; Mamsen et al., 2012). Here, they are exposed to signals from the somatic precursor cells which directs the differentiation of the PGCs towards either male (gonocytes) or female (oogonia) fate. The somatic cell population in the developing gonads consist mainly of the supporting- and steroidogenic cell lineages. The supporting cells are precursors of Sertoli cells (testis) and granulosa cells (ovary), while the steroidogenic cells develops into Leydig cells (testis) or theca cells (ovary).

Similar to mice, the sex-specific differentiation of human bipotential gonads is initiated by a fate decision in the supporting cells which directs the development of the gonads resulting in physiological events specific for either the testis or ovary. SRY is the main determinant of human testicular development and translocation of Y-chromosome fragments containing the *SRY* gene accounts for 90% of 46,XX testicular DSDs (Knarston et al., 2016). Similarly, approximately 20% of all 46,XY DSD patients have loss-of-function mutations in the coding region of *SRY* (Koopman, 2016). Expression of *SRY* in the supporting cells from around GW 6 promotes the upregulation of SOX9 and the initiation of Sertoli cell differentiation (Sinclair et al., 1990; Hanley et al., 2000). Consequently, loss of function mutations in *SOX9* or in enhancer elements upstream of the *SOX9* gene is thus associated with 46,YX sex reversal (Foster et al., 1994; Croft et al., 2018). FGF9 also appears to cooperate with SOX9 in the establishment of human Sertoli cell identity since loss-of-function of the FGF9 receptor FGFR2 leads to 46,XY DSD with sex reversal (Bagheri-Fam et al., 2015). Following the initial differentiation of the Sertoli cells, morphological changes including the formation of seminiferous cords are observed from GW 7–8, which is accompanied by the expression of AMH (Ostrer et al., 2007). AMH is an important factor that ensures regression of the Müllerian ducts in male fetuses and reproductive tract dysfunctions are observed in 46,XY individuals with mutations that affects the function of the *AMH* gene or the AMH receptor *AMHR2* (Imbeaud et al., 1994; Mazen et al., 2017). The Sertoli cells promote differentiation of fetal Leydig cells from the steroidogenic cell lineage through paracrine signalling. The two factors believed to be involved in the differentiation of fetal Leydig cells though paracrine signalling is DHH and PDGF. *DHH* is secreted by Sertoli cells, while the receptors *PTCH1* and *PTCH2* mediating the effects of DHH are expressed in Leydig cell precursors based on single cell RNA sequencing data from (Li et al., 2017) viewed in the ReproGenomicsViewer (Darde et al., 2019). Loss of function mutations in *DHH* is associated with 46,XY DSD ranging from partial to complete gonadal dysgenesis with the presence of Müllerian structures (Rothacker et al., 2018; Pachernegg et al., 2021). Conversely, mutations in the PDGF system has not been associated with DSD but instead found in various cancers and fibrotic diseases (Guérit et al., 2021). However, little is currently known about the precise role of DHH and PDGF in human fetal testis development and its involvement in Leydig cell differentiation and function. Importantly, the fetal Leydig cells produce testosterone already from approximately GW 7–8 (Albalushi et al., 2019; Savchuk et al., 2019) and Insulin-like Factor 3 (INSL3) from GW 8–9 (Ben Maamar et al., 2015). The production of both testosterone and INSL3 is essential for masculinisation of the foetus and in particular in the development of the male reproductive system including the differentiation of the Wolffian duct and testicular descent (Bay et al., 2007).

Together the paracrine factors and steroid hormones secreted in the human fetal testes further promotes the sex-specific development of the somatic cell lineages and the establishment of the somatic niche which supports the germ cell population, including germ cell proliferation and the differentiation of gonocytes to pre-spermatogonia. This differentiation to pre-spermatogonia occurs asynchronously in the human fetal testis from approximately GW 16–20 and is associated with the gradual downregulation of pluripotency factors and upregulation of e.g. MAGE-A4 (Gaskell et al., 2004; Hoei-Hansen et al., 2004; Rajpert-De Meyts et al., 2004). The differentiation of pre-spermatogonia occurs in a more synchronous manner in mouse testes and since the expression of the pluripotency factor OCT4 also persist in adult mouse spermatogonia, this constitutes an important difference in germ cell development between mice and humans (Pesce et al., 1998).

The understanding of signalling directing sex-specific differentiation of the somatic cell populations in human fetal ovaries are currently less detailed. From studies in mice, it is suggested that absence of SRY in combination with initiation of the WNT/β-catenin signalling pathway promotes the differentiation of the supporting cell lineage to granulosa cells. At the time of early human gonad development, *WNT4* appears to be similarly expressed in testes and ovaries, while *RSPO1* is ovary-specifically expressed (Tomaselli et al., 2011; Mamsen et al., 2017). Despite this, *WNT4* loss-of-function mutations in 46,XX individuals has been reported to result in virilization and lack of Müllerian structures, development of ovotestis or complete female-to-male sex reversal (Biason-Lauber et al., 2004; Mandel et al., 2008). Detailed functional analysis of three *WNT4* mutations in 46,XX individuals with Müllerian duct abnormalities and hyperandrogenism suggested that WNT4 is involved in ovarian development through repression of androgen biosynthesis (Biason-Lauber et al., 2007; Philibert et al., 2008, 2011). Likewise, loss-of-function mutations in *RSPO1* was reported to result in either complete female-to-male sex reversal of a 46,XX individual (Parma et al., 2006) or 46,XX ovotesticular DSD (Tomaselli et al., 2008; Naasse et al., 2017). In accordance, mutations in *ZNRF3* that normally antagonizes WNT signalling have been associated with male-to-female sex reversal in four 46,XY individuals (Harris et al., 2018). Together this suggests an important role for WNT4 and RSPO1 also in human fetal ovary development.

The granulosa cell factor FOXL2 is also considered to be important in ovary development and recent single cell RNA sequencing studies of early human embryonic and fetal ovaries has shown the presence of several granulosa cell subpopulations (Vento-Tormo et al., 2021, preprint), two of these as early pre-granulosa cells with a FOXL2-positive and a FOXL2-negative population, similar to what has been demonstrated in mice (Rastetter et al., 2014; Zheng et al., 2014; Niu and Spradling, 2020). However, loss-of-function mutations in *FOXL2* in 46,XX individuals resulting in masculinization or female-to-male sex reversal have not been reported (Bashamboo and McElreavey, 2015). Instead primary ovarian insufficiency, which is the depletion of the follicular pool in adult women before 40 years of age has been described for FOXL2 heterozygous mutations (Harris et al., 2002). Although this suggest that FOXL2 is not the main determinant of early ovarian sex-specific differentiation but rather has an important function in the maintenance of granulosa cell fate after its establishment, homozygous mutations in *FOXL2* have not to our knowledge been reported. Interestingly, a homozygous FOXL2 mutation induced by mutagenesis have been described in goats where it is was associated with female-to-male sex reversal (Boulanger et al., 2014). Thereby emphasising that the role of FOXL2 in early ovarian differentiation is not yet fully understood. In line with the notion that FOXL2 may not be the main determinant of early ovarian development, transcriptional analysis of human fetal ovaries revealed expression of *RSPO1* prior to the expression of *FOXL2* (Lecluze et al., 2020).

The sex-specific differentiation of the supporting cells into either Sertoli cells or granulosa cells is however not fixed once initially established. Thus, continuous repression of the opposite pathway is crucial for proper sex development. In humans, loss of function mutations in *DMRT1* results in varying degrees of sex reversal and 46,XY DSD (Vinci et al., 2007; Murphy et al., 2015), and is thus suggested to be important for sex-specific differentiation of the testes. However, whether DMRT1 directly repress FOXL2 expression and hence ovarian fate, thereby contributing to testes differentiation as it has been shown in mice (Matson et al., 2011; Lindeman et al., 2015), remain unanswered.

In contrast to the fetal Leydig cells which can be detected in the testes from GW 8–9, differentiation of theca cells from the steroidogenic cell lineage in fetal ovaries occurs much later during gestation. The first theca cells are observed in late third trimester once folliculogenesis has been initiated (Konishi et al., 1986), and here theca cells surround the growing follicles from the secondary stage and ensure the production of steroid hormones important to sustain folliculogenesis.

Oogonia, which are the undifferentiated type of germ cells present in the fetal ovary, progress through series of mitotic divisions once they have entered the developing gonads (Sørensen et al., 2011). Notably, already from GW 9 the number of oogonia are approximately nine-fold higher than the number of gonocytes in the fetal testis due to their high proliferation rate (Bendsen et al., 2003, 2006). From around GW 10 a subset of oogonia initiate meiosis (Gondos et al., 1986; Bendsen et al., 2006; Le Bouffant et al., 2010; Jorgensen et al., 2012; Frydman et al., 2017), while another subpopulation continue to proliferate until around GW 19–20 (Hartshorne et al., 2009; Rosario et al., 2016). The mechanism by which meiosis is initiated in human fetal ovaries appears to in part involve the action of RA and the upregulation of *STRA8* (Le Bouffant et al., 2010; Childs et al., 2011; Jorgensen et al., 2015), although the signalling and regulation involved is not fully understood. The initiation of meiosis marks the transition from oogonia to oocytes, which is associated with downregulation of pluripotency factors (Robinson et al., 2001; Hoei-Hansen et al., 2004; Rajpert-De Meyts et al., 2004; Stoop et al., 2005; Kerr et al., 2008; Byskov et al., 2011; Childs et al., 2012), and up-regulation of germ cell and meiosis markers (Anderson et al., 2007; Le Bouffant et al., 2010; Childs et al., 2011). After initiation of meiosis, the oocytes are enclosed by squamous granulosa cells to form the first follicles which are reported to appear in the second trimester, although the exact timing vary a little between studies (Forabosco and Sforza, 2007; Fowler et al., 2009; Lundgaard Riis et al., 2021).

## Lessons From *ex vivo* Culture of Human Fetal Gonads

Experimental studies investigating signalling pathways involved in human fetal gonad development are relatively few, mainly due to the limited access to human fetal gonad tissue and the challenges of long-term culture. However, in recent years several *ex vivo* culture models have been established, which allows functional studies of early human fetal gonad development. While several studies have focused on assessing the effect of endocrine disrupting chemicals, including various analgesics (Lambrot et al., 2009; Tartarin et al., 2012; Mazaud-Guittot et al., 2013; Ben Maamar et al., 2017; Gaudriault et al., 2017), other studies have attempted to elucidate the mechanisms of normal human fetal gonad development and function through manipulation of selected signalling pathways (Le Bouffant et al., 2010; Childs et al., 2011; Jorgensen et al., 2015; Poulain et al., 2015; Frydman et al., 2017; Jørgensen et al., 2018; Macdonald et al., 2018; Harpelunde Poulsen et al., 2019).

**FIGURE 1:**
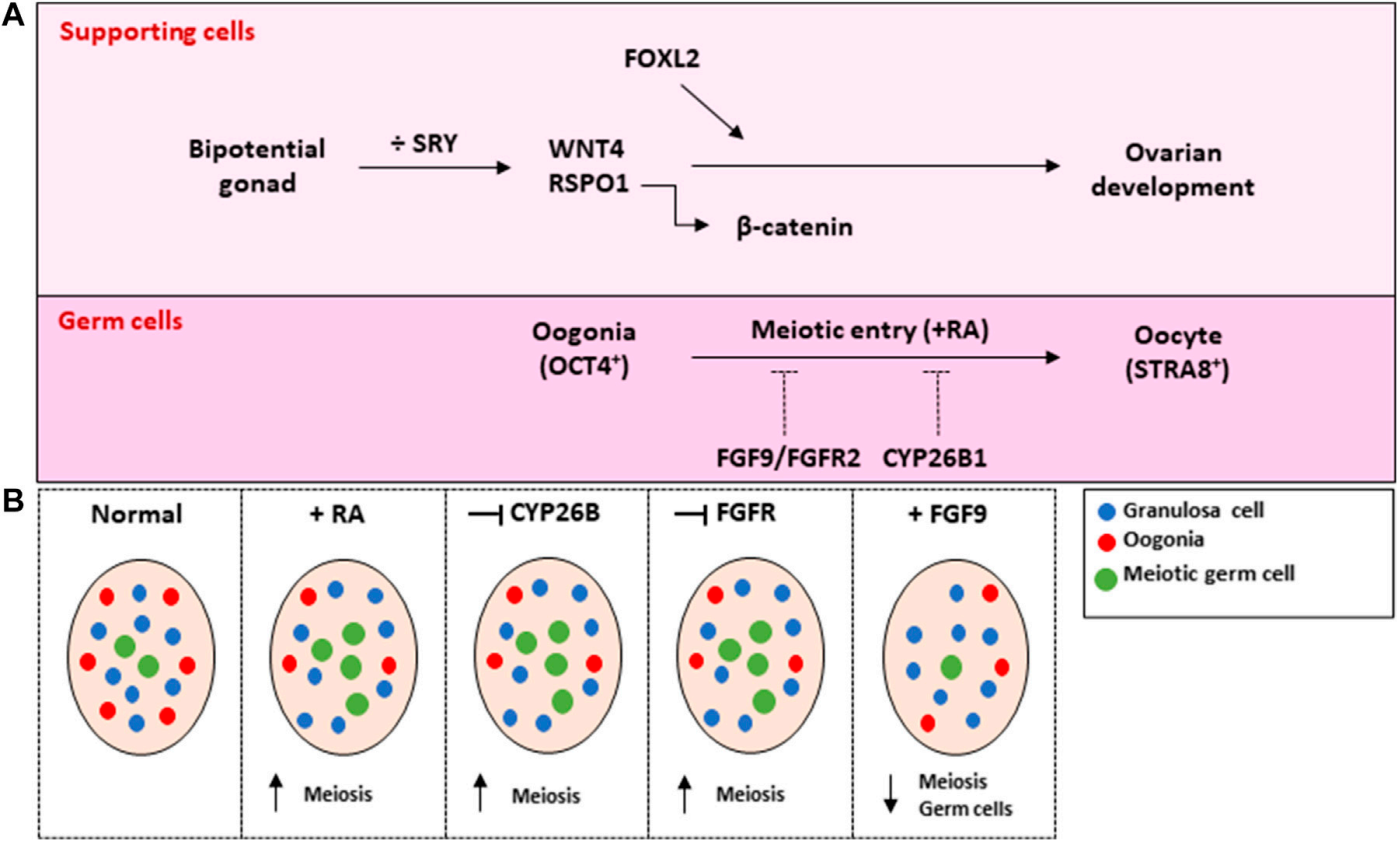
Current understanding of the sex-specific differentiation of human fetal ovaries based on information from mouse models, patients with Disorders of sex development and *ex vivo* culture models. **(A)** In the bipotential gonad of a XX (female) embryo, absence of SRY and upregulation of pro-ovarian factors WNT4 and RSPO1 initiates the differentiation of the supporting cells to granulosa cells and thus ovarian fate through stimulation of the *β*-catenin signalling pathway. FOXL2 also plays an important role in the establishment and maintenance of granulosa cell identity. The somatic niche established by the granulosa cells promotes germ cell commitment to the female developmental pathway by supporting proliferation of oogonia as well as initiation of meiosis in a subset of oogonia through at least to some extend the actions of retinoic acid (RA) and expression of STRA8. This process is actively repressed in another subset of oogonia by FGF9 and CYP26B1. **(B)** Illustration and summary of findings from the manipulation of selected signalling factors in *ex vivo* cultured human fetal ovaries. References to the studies included in the figure can be found in the text.

**FIGURE 2:**
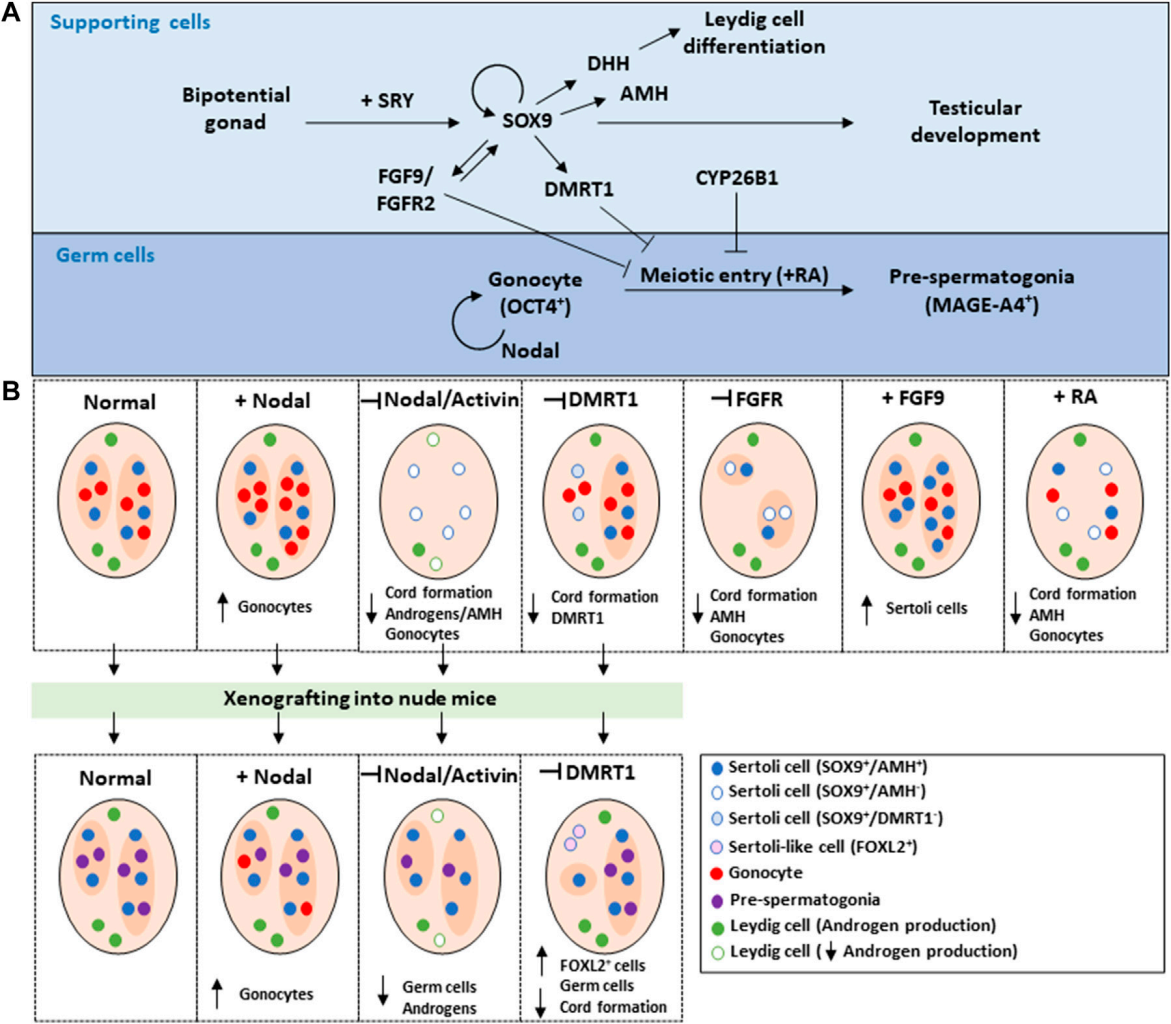
Current understanding of the sex-specific differentiation of human fetal testes based on information from mouse models, patients with Disorders of sex development and *ex vivo* culture models. **(A)** In the bipotential gonad of a XY (male) embryo, SRY is upregulated in the supporting cells and induce the expression of SOX9 which together with FGF9/FGFR2 promote the differentiation of the supporting cell lineage into Sertoli cells. AMH expressed by the Sertoli cells further supports male development of the foetus by ensuring regression of the Müllerian ducts. The Sertoli cells stimulates the differentiation of the steroidogenic cell lineages (Leydig cells) through paracrine signalling mediated by DHH. Additionally, the Sertoli cells directs the germ cell commitment to the male developmental pathway by repressing meiotic entry through the actions of CYP26B, FGF9/FGFR, and DMRT1, while Nodal signalling is involved in regulating germ cell pluripotency and thus the regulation of gonocyte to pre-spermatogonia differentiation. **(B)** Illustration and summary of findings from the manipulation of selected signalling factors in *ex vivo* cultured human fetal testes, which in some cases included manipulations in *ex vivo* culture of human testes followed by xenografting into nude mice to examine long-term effects. References to the studies included in the figure can be found in the text.

Several studies have examined the regulation of meiotic entry in early human fetal gonad development (Le Bouffant et al., 2010; Childs et al., 2011; Jorgensen et al., 2015; Frydman et al., 2017; Harpelunde Poulsen et al., 2019). It was shown that meiosis is initiated from GW 10 in human fetal ovaries and that addition of exogenous RA to *ex vivo* cultured ovaries results in an increased number of meiotic cells (Le Bouffant et al., 2010) (Figure 1). This was subsequently confirmed in an independent study (Jorgensen et al., 2015). Together these results suggest that RA at least to some extend may be involved in inducing meiosis in human fetal ovaries although this does not exclude the existence of additional meiosis inducing substances. Conversely, in the human fetal testis meiotic entry is actively repressed in the germ cells until puberty, which constitutes an important sex-specific difference that are in place to prevent premature initiation of meiosis until spermatogenesis can be supported by the somatic niche. Accordingly, treatment of *ex vivo* cultured fetal testis with RA resulted in a reduced number of gonocytes as well as a reduced number of proliferative cells indicating that abnormal RA signalling may have consequences for germ cell survival (Jorgensen et al., 2015) (Figure 2). The involvement of FGF9 signalling in meiosis regulation have also been examined in *ex vivo* cultured human fetal gonad development. As mentioned above FGF9 is testis-specifically expressed in mice after initiation of gonadal sex differentiation and is involved in preventing premature meiotic entry in male germ cells. In contrast, FGF9 was expressed in both human fetal testes and ovaries at transcriptional and protein level without a clear sex-specific difference (Frydman et al., 2017; Harpelunde Poulsen et al., 2019). In *ex vivo* cultured fetal ovaries, treatment with recombinant FGF9 resulted in a reduced number of meiotic cells, while inhibition of FGFR signalling significantly increased the number of meiotic cells (Frydman et al., 2017) (Figure 1). In an independent study, inhibition of FGFR signalling resulted in reduced germ cell survival and similar to (Frydman et al., 2017), culture of fetal ovaries with FGF9 resulted in a reduced number of meiotic cells (Harpelunde Poulsen et al., 2019) (Figure 1). Together this suggests that FGF9 may prevent a subset of oogonia from initiating meiosis and thereby be involved in regulating the asynchronous pattern of meiotic entry observed in the human fetal ovary. Accordingly, no clear sex-dimorphic expression pattern of the RA-degrading enzyme CYP26B1 was observed in human fetal gonads (Le Bouffant et al., 2010; Childs et al., 2011), which is different from mice where the expression of *Cyp26b* is testis-specific from E11.5–12.5 (Kashimada et al., 2011). Inhibition of CYP26B1 in human fetal ovaries resulted in an increased number of meiotic cells, indicating that CYP26B1 may play a role in regulating meiotic entry in the human fetal ovary (Frydman et al., 2017) (Figure 1). Importantly, these results emphasize a difference between mice and humans in the regulation of meiotic entry and thus highlights the complexity of the signalling involved as well as the need for a cautious translation of findings from studies in mice to humans.

Another important aspect of normal germ cell differentiation during fetal development is the regulation of pluripotency factor expression. Failure to downregulate pluripotency factors may give rise to the development of precursor cells for testicular germ cell cancer, termed germ cell neoplasia *in situ* (GCNIS) (Rajpert-De Meyts, 2006; Rajpert-De Meyts et al., 2016), which does not develop in mice. Interestingly, our previous study examining the role of Nodal and Activin signalling in human fetal testes *ex vivo*, demonstrated involvement of the Nodal pathway in the regulation of germ cell pluripotency and thus in the differentiation of gonocytes to pre-spermatogonia. Nodal stimulation resulted in a prolonged presence of OCT4-positive gonocytes within the human fetal testes (Jørgensen et al., 2018) (Figure 2). Conversely, inhibition of either Nodal or Activin A signalling resulted in a reduced number of gonocytes. Together indicating a role for Nodal signalling in regulating pluripotency factor expression in human fetal gonocytes which is in accordance with the role of Nodal signalling in mice (Souquet et al., 2012; Spiller et al., 2012).

The sex-specific differentiation of human fetal gonads are directed by the supporting cell populations, but only few studies have focused on examining these regulatory networks in *ex vivo* cultures. In a recent study, the role of FGF9 in human fetal testis development was examined by inhibiting FGFR signalling (Harpelunde Poulsen et al., 2019). This resulted in pronounced alterations in the somatic niche, including reduced AMH expression, a reduction in the number of SOX9-positive Sertoli cells and altered seminiferous cord structure (Harpelunde Poulsen et al., 2019) (Figure 2). Conversely, stimulation of FGF9 signalling increased the number of Sertoli cells in *ex vivo* cultured human fetal testes (Harpelunde Poulsen et al., 2019) (Figure 2). Together suggesting that FGF9 signalling is involved in promoting Sertoli cell differentiation during testis development similar to its role in mouse testis.

Manipulation of another important Sertoli cell factor DMRT1 has also been examined in human fetal testes cultures. DMRT1 is known to be a negative regulator of meiotic entry and to maintain Sertoli cell fate in mice while in humans, DMRT1 appears to be important for sex-specific differentiation of the testes. Despite this, the mechanism by which DMRT1 contribute to the early differentiation of fetal testis remain poorly understood. Lentivirus mediated knockdown of *DMRT1* in *ex vivo* cultured fetal testis tissue was followed by xenografting into nude mice in order to examine long-term effects. Loss of *DMRT1* expression resulted in focal dysgenesis with loss of seminiferous cord structures, loss of germ cells and ectopic expression of FOXL2 in areas with dysgenesis (Macdonald et al., 2018) (Figure 2). This indicates focal trans-differentiation of the Sertoli cells to granulosa-like cells similar to observations from *DMRT1* knockdown in mice (Matson et al., 2011; Minkina et al., 2014; Lindeman et al., 2015), and thus suggests that DMRT1 expression is essential for maintenance of testicular fate also in humans. The role of another member of the DMRT family, DMRT5, in human fetal ovaries was examined by knockdown of expression during xenografting into nude mice by using siRNA interference. Inhibition of DMRT5 was associated with impaired expression of pre-meiotic germ cell markers, a reduced percentage of meiotic cells and an increase in undifferentiated germ cells (Poulain el al., 2015), thereby indicating a role of DMRT5 in germ cell development in the human ovary.

In the study examining the role of Nodal signalling in the regulation of germ cell pluripotency, we also found that simultaneous inhibition of Nodal and Activin signalling in human fetal testes results in effects on the somatic niche, including disrupted seminiferous cord formation, reduced AMH expression and secretion as well as reduced production of androgens (Jørgensen et al., 2018) (Figure 2). Furthermore, treatment of human fetal testes with the Nodal-specific inhibitor Lefty resulted in disrupted seminiferous cord formation and AMH expression and secretion, while treatment with the Activin-specific inhibitor Follistatin overall did not affect testicular morphology or expression of somatic cell markers. Together suggesting that inhibition of Nodal signalling accounted for the majority of the effects on the somatic cells observed following the simultaneous inhibition. Of notice, treatment of human fetal testes with the proposed meiosis-regulator RA also resulted in impaired seminiferous cord structures and reduced AMH expression (Jorgensen et al., 2015) (Figure 2). Thereby indicating that abnormal signalling between germ cells and somatic cells during fetal testis development may have consequences beyond the impact on germ cells including effect on testicular morphology, expression of somatic cell lineage markers, and somatic cell function.

## Perspectives

Deciphering the regulatory networks and mechanisms behind sex-specific differentiation of human fetal gonads is a continuous challenge with numerous questions remaining unanswered. In particular, WNT4/β-catenin signalling remains largely unexplored in human fetal ovaries, while the mechanism by which DHH exert the crosstalk between the Sertoli cells and Leydig cells, and its importance in Leydig cell differentiation also remains largely unknown. Additionally, the conflicting evidence from mice regarding the role of RA in the initiation of meiosis highlight that several unanswered questions about the regulation of meiotic entry and role of RA in human fetal ovaries remains to be elucidated. Currently much of the knowledge about sex-specific gonadal development originates from knockout mice models, but due to the existence of some species-specific differences—a cautious translation of findings from studies in mice to humans is essential. Information from DSD patients may also be useful in identifying genes and factors that are important to ensure normal sex-specific gonad development, although there are limited possibilities to examine the underlying mechanisms invloved. In particular, the fetal origin of gonadal phenotypes observed in DSD patients represents a challenge. Therefore, established *ex vivo* models of human fetal gonads may provide a useful tool to assess the consequences resulting from manipulation of specific signalling pathways. However, *ex vivo* culture models do not recapitulate the *in vivo* situation and thus, research examining sex-specific gonadal development in humans must continue to be based on multiple approaches. Combining information from mouse models, DSD patients and *ex vivo* culture models of human fetal gonads may currently be the best strategy to understand the signalling involved in directing the sex-specific differentiation of human fetal gonads and the development of fetal testes and ovaries, respectively.
